# Electrophysiological Recording in the *Drosophila* Embryo

**DOI:** 10.3791/1348

**Published:** 2009-05-21

**Authors:** Kaiyun Chen, David E. Featherstone, Kendal Broadie

**Affiliations:** Department of Biological Sciences, University of Illinois at Chicago; Department of Biological Sciences, Vanderbilt University

## Abstract

*Drosophila* is a premier genetic model for the study of both embryonic development and functional neuroscience. Traditionally, these fields are quite isolated from each other, with largely independent histories and scientific communities. However, the interface between these usually disparate fields is the developmental programs underlying acquisition of functional electrical signaling properties and differentiation of functional chemical synapses during the final phases of neural circuit formation. This interface is a critically important area for investigation. In *Drosophila*, these phases of functional development occur during a period of <8 hours (at 25°C) during the last third of embryogenesis. This late developmental period was long considered intractable to investigation owing to the deposition of a tough, impermeable epidermal cuticle. A breakthrough advance was the application of water-polymerizing surgical glue that can be locally applied to the cuticle to enable controlled dissection of late-stage embryos. With a dorsal longitudinal incision, the embryo can be laid flat, exposing the ventral nerve cord and body wall musculature to experimental investigation. Whole-cell patch-clamp techniques can then be employed to record from individually-identifiable neurons and somatic muscles. These recording configurations have been used to track the appearance and maturation of ionic currents and action potential propagation in both neurons and muscles. Genetic mutants affecting these electrical properties have been characterized to reveal the molecular composition of ion channels and associated signaling complexes, and to begin exploration of the molecular mechanisms of functional differentiation. A particular focus has been the assembly of synaptic connections, both in the central nervous system and periphery. The glutamatergic neuromuscular junction (NMJ) is most accessible to a combination of optical imaging and electrophysiological recording. A glass suction electrode is used to stimulate the peripheral nerve, with excitatory junction current (EJC) recordings made in the voltage-clamped muscle. This recording configuration has been used to chart the functional differentiation of the synapse, and track the appearance and maturation of presynaptic glutamate release properties. In addition, postsynaptic properties can be assayed independently via iontophoretic or pressure application of glutamate directly to the muscle surface, to measure the appearance and maturation of the glutamate receptor fields. Thus, both pre- and postsynaptic elements can be monitored separately or in combination during embryonic synaptogenesis. This system has been heavily used to isolate and characterize genetic mutants that impair embryonic synapse formation, and thus reveal the molecular mechanisms governing the specification and differentiation of synapse connections and functional synaptic signaling properties.

**Figure:**
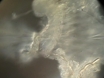


## Protocol

### Part 1: Equipment and Supplies

Electrophysiological recording from *Drosophila* embryos first requires proficiency in embryonic dissection techniques, which are described in another JoVE video.
Electrophysiological recording from *Drosophila* embryos utilizes standard patch clamp recording configurations.  Patch clamp recording equipment and software suitable for many other preparations is also suitable for recording from *Drosophila* embryos.  Because *Drosophila* embryos are glued to a cover slip during dissection, the recording chamber should accept cover slips.  The thickness of the preparation and manner in which it is mounted to the coverslip requires an upright microscope with excellent DIC optics and a long working distance lens.
Two types of bath solutions are used for electrophysiological recording; 1) “standard” (Jan and Jan, 1976a) or “modified standard” (Broadie, 2000) salines, based on solutions commonly used for recording in other invertebrate systems, and 2) “haemolymph-like” (HL) salines (Stewart et al 1994), a compromise between the standard saline and the ionic concentrations measured in the *Drosophila* haemolymph. It should be noted that none of these salines accurately mimic the ionic concentrations measured in the haemolymph, which are unfavorable for recording  (Broadie, 2000). Standard saline contains (in mM): 135 NaCl, 5 KCl, 4 MgCl_2_, 1.8 CaCl2, 72 sucrose, 5 TES, pH 7.2.

### Part 2: Recording Configuration

For physiological experiments, the dissected embryo preparation is placed in a small plexiglass recording chamber (<0.5 ml) and viewed in transmitted light with an upright compound microscope fitted with differential interference contrast (Nomarski) optics and a long-working distance 40-100X (typically 40X) water-immersion objective (Broadie and Bate, 1993a). Recordings are typically made below room temperature (16-22°C), with increasing difficulty at elevated temperatures (22+°C).  We achieve this by cooling the room rather than cooling the preapartion.
Patch pipettes can be pulled from a variety of fiber-filled glasses (e.g. borosilicate glass, 1 mm outer diameter) and the tips should be fire-polished to final resistances of 5-10 MΩ. A chloride-silver ground wire is placed in the bath. Signals are amplified using a patch-clamp amplifier (e.g. Axopatch-1D, Axon Instruments), filtered with an 8-pole Bessel filter at 2-10 Hz, and either sampled on-line or stored digitally for later analysis.
Conventional patch-clamp recordings are most often made at 18°C with patch pipettes pulled from fiber-filled borosilicate glass (tips heat polished to ~1 µm i.d.). Current records are made from identified multinucleate muscle fibers in voltage-clamp (standard holding potentials –60 to –80 mV) (Broadie and Bate, 1993a-d; Broadie et al., 1997; Fergestad et al., 1999; Featherstone et al., 2000).  The patch pipette solution contains (in mM): 120 KCl, 20 KOH, 4 MgCl_2_, 0.25 CaCl_2_, 5 EGTA, 4 Na_2_ATP, 36 Sucrose, and 5 TES, buffered at pH 7.2. Recording electrodes are back-filled.

### Part 3: Muscle Recording

In principle, any embryonic muscle may be recorded from in this preparation.  In practice, most recording has concentrated on the large, ventral longitudinal muscles in the inner-most muscle layer (Bate, 1990; Broadie and Bate, 1993a-c). Records have also been made from the dorsal and lateral muscles of this muscle layer (Kidokoro and Nishikawa, 1994; Auld et al. 1995; Nishikawa and Kidokoro, 1995; Baumgartner at al. 1996). However, most experiments focus on the group of four longitudinal, ventral muscles (6,7,12,13), particularly muscle 6 in the anterior abdomen (A2-A3).
No enzymatic treatment is required prior to muscle recording in young embryos (<16 hrs AEL). However, a muscle sheath develops in the later embryo (>16 hrs AEL) and must be removed with collagenase (collagenase type IV, 1 mg/ml in divalent cation-free saline, 0.5-2 min at RT) prior to patch recording. Following enzyme treatment, seal resistances on the muscle are typically >1 GΩ.
Patch-clamp recordings can be made from the embryonic muscles using a number of standard techniques (Broadie and Bate 1993a-c; Broadie et al. 1994, 1995, 1997; Nishikawa and Kidokoro, 1995). Any standard patch-clamp variations -- cell-attached, inside-out, outside-out -- can be employed to record single channel activity in the muscle or isolated muscle membrane patches.  Single glutamate receptor channel activity (~12 pA at -60 mV) are generally observable on the falling phase of spontaneous excitatory junction currents (sEJCs) recorded in whole cell mode.
Most recording is done in whole-cell recording configuration, achieved easily from the cell-attached state with slight suction or an electrical “buzz”. The muscles can be recorded from either in voltage-clamp (typical holding potentials of -60 to -80 mV) or current-clamp configurations. Muscles in younger embryos (<13 hrs AEL) cannot be effectively voltage-clamped owing to extensive coupling between the embryonic myotubes (Broadie and Bate, 1993a); this is usually not a problem during later (14+ hrs AEL) developmental stages (Ueda and Kidokoro, 1996).
Nystatin-perforated patch-clamp recording can be used to verify records obtained with standard whole-cell techniques (Broadie and Bate, 1993a). The perforation solution is made as follows: 10 mg pluronic F-127 (Molecular Probes p-1572) is dissolved in 20 ml DMSO, and 10 mg Nystatin dissolved in this solution to make the stock. 100 μl of the stock is added to 5 ml filtered pipette solution to make the recording solution. Whole-cell currents are usually obtained <3 mins after seal formation. Series resistance is markedly increased in most instances and cells can develop an increased leakage current (Broadie and Bate, 1993a).
The whole-cell voltage-gated current in mature embryonic (and larval) muscle is composed of five prominent components (Wu and Haugland, 1985; Zagotta et al. 1988; Broadie and Bate, 1993b): an inward calcium current (I_Ca_) and four outward potassium currents (Tsunoda and Salkoff, 1995), including two fast, inactivating currents, voltage-gated (I_A_) and calcium-dependent (I_CF_), and two delayed, non-inactivating currents, voltage-gated (I_K_) and calcium-dependent (I_CS_). Ion-substitution experiments can be used to dissect these currents from the whole-cell response during voltage-clamp (Broadie and Bate, 1993b).

### Part 4: Neuromuscular Junction Stimulation and Recording

The most common and reliable method of NMJ stimulation uses a glass suction electrode on the segmental peripheral nerve. Suction electrodes can be pulled from a variety of fiber-filled electrode glass (1-1.5 μm outer diameter) and should be fire-polished to achieve the desired configuration (~5 μm inner diameter). A small segment (<50 μm) of the appropriate segmental motor nerve is drawn into the pipette with gentle suction to form a tight seal.
It is common to stimulate the intact nerve by drawing in a loop of the nerve near the CNS exit point. This approach allows easy stimulation of the intact preparation, but has the disadvantage that spontaneous CNS-evoked activity can occur superimposed on the applied stimulation paradigm. An alternative is to stimulate the cut motor nerve. This can be achieved if the CNS is removed, although this procedure tends to stretch the nerve and may cause damage. The nerve may also be cut with a sharp glass microelectrode, but this procedure is tedious and difficult. It is recommended that records be made from the intact preparation.
Stimulation can be applied with a variety of paradigms. Brief stimulation (0.2-1 msec) works best and stimulation intensity (usually 1-5 V) will depend on the suction configuration and must be experimentally determined for each cell. Suprathreshold stimulation of the motor nerve is best achieved by setting the stimulus strength slightly above (+1-2 volts) threshold.  Evoked NMJ responses are recorded from the patch-clamped muscle as described above. The shock artifact can be substantially decreased with the use of an isolated virtual ground.
A less reliable alternative to the standard suction electrode nerve stimulation is direct stimulation of the central nervous system (Nishikawa and Kidokoro, 1995). A microelectrode filled with 3-4 M KCl  (or KAc) is inserted into the middle of the ventral ganglion and positive pulses of ~2 microamp in intensity and ~2 msec in duration are delivered (Dietcher et al. 1998). The electrode tip should be in the neuropil nearest the hemisegment where stimulation is desired.  Synaptic transmission is recorded in the patch-clamped muscle in the standard configuration.
The embryonic NMJ is accessible to whole-cell patch-clamp recording throughout its differentiation. For excitatory junctional current (EJC) recordings, signals are recorded with an standard patch-clamp amplifier, filtered (usually at 1-2 KHz), converted to a digital signal using an analog-to-digital interface, and acquired with a computer using appropriate software.

### Part 5: Neuromuscular Junction Chemical and Drug Applications

Any small, charged molecule can be effectively iontophoresed onto the embryonic NMJ (Aravamudan et al, 1999; Fergestad et al. 1999, 2001). Iontophoretic pipettes can be pulled for specific configurations, with applications for variable periods, using pulses of negative or positive current. It is vital to prevent leakage between pulses with an appropriate opposing backing current (Broadie et al. 1993d; Featherstone et al. 2002, 2005; Rohrbough et al. 2007). The magnitude of this backing current will vary with pipette configuration and should be experimentally determined.
Many experiments have been done with the NMJ neurotransmitter, L-glutamate (Jan and Jan, 1976b), using a 100mM stock solution (Monosodium salt; pH 8-9)(Broadie and Bate, 1993c). High resistance iontophoretic pipettes (100-200 MΩ) have been used for glutamate receptor (gluR) mapping (Broadie and Bate, 1993d), and lower resistance pipettes (20-50 MΩ) used to estimate the total gluR response (Broadie et al. 1995, 1997; Featherstone et al. 2002, 2005; Rohrbough et al. 2007). Short pulses (0.1-1 ms) of negative current (~10 nA) work well, with a small, positive backing current to prevent leakage.
Non-charged and charged molecules can be applied by pressure ejection (Fergestad and Broadie, 2001; Featherstone et al. 2001). For application, a pressure ejection pipette (<5 μm inner diameter) containing the solution is positioned close (<10 μm) from the nerve terminal. The solution to be ejected can be applied using a picospritzer (5-10 psi) or other pressurized air source (Featherstone et al., 2002). During prolonged experiments, the recording chamber (volume <0.5 ml) should be perfused continuously (0.1 - 0.2 ml/sec) with normal bath saline.
Many experiments have been done with hyperosmotic saline to trigger fusion of synaptic vesicles (Broadie et al. 1995; Aravamudan, et al., 1999; Fergestad et al., 1999; Featherstone, et al., 2000, 2002). Other experiments have been done with toxins, such as argiotoxin 636 against glutamate receptors and black widow spider venom containing latroinsectotoxin (Broadie et al. 1995).

### Part 6: Complementary Neuromuscular Junction Vital Imaging Techniques

Synaptic vesicle kinetics may be monitored with fluorescent lipophilic stryl dyes (Fergestad and Broadie, 2001; Rohrbough et al. 2004). The distribution of vesicles can be directly observed, and rates of endocytosis and exocytosis measured. Several variants have been made with different fluorescent properties (FM1-43, fluorescein-like; FM4-64, rhodamine-like).  Modifications of the original dye have yielded products with altered hydrophobic properties, and therefore with different washout kinetics; e.g., FM2-10 dissociates much faster from the membrane.
To label the embryonic NMJ with stryl dyes, 10 µM FM1-43 can be loaded for 1-5 minutes using elevated extracellular potassium (50-90 mM) (Fergestad and Broadie, 2001).  A more physiological approach may be achieved via stimulation by a nerve suction electrode. Preparations are then washed repeatedly in Ca^2+^-free buffer for 5 minutes, and retained fluorescence (representing dyed synaptic vesicles) measured.  To measure exocytosis, one can unload the dye using either elevated potassium or nerve electrical stimulation.
Fusion proteins with GFP variants have been produced allowing multicolor labeling of the embryonic NMJ synapse (Brand, 1999; Zhang et al. 2002). Many transgenics expressing GFP-tagged proteins are available including general membrane markers (e.g. mCD8-GFP), postsynaptic markers (e.g. Shaker-GFP, GluRIIA-GFP), cytoskeletal (e.g. actin-GFP, moesin-GFP, tubulin-GFP) and mitochondrial markers (e.g. Cytochrome C-GFP) and synaptic vesicle markers (e.g. synaptobrevin-GFP, synaptotagmin-GFP; Zhang et al., 2002).
GFP reporters label compartments that are highly dynamic and can be used to assess developmental features or mutant phenotypes (Featherstone and Broadie, 2000). Bleaching and then observing the recovery of the fluorescence (FRAP) has been shown to be feasible, at least at the larval *Drosophila* NMJ (Zhang et al., 2002; Yan and Broadie, 2007).  A word of caution, however: some of these markers do not fully rescue null mutants and may even alter normal NMJ physiology.

### Part 7: Recording from Central Motor Neurons

Many embryonic neurons can be individually identified based on soma position and projection morphology (Bate and Martinez-Arias, 1993; Goodman and Doe, 1993; Landgraf et al 1997).  Further, the GAL4/UAS system (Brand and Perrimon, 1993) can target GFP expression to visualize identifiable neuron populations (Baines et al., 1998). Recording studies to date have concentrated on a group of five superficial, dorsal neurons within the embryonic ventral nerve cord (VNC); four motor neurons (aCC and RP1-4) and one interneuron pCC (Baines et al., 1998, 1999, 2001; Rohrbough and Broadie, 2002; Featherstone et al. 1995).
Cell bodies of identified neurons are accessible to whole-cell patch-clamp recording electrodes. The VNC neurolemma sheath must first be removed with brief focal treatment with protease (Baines and Bate, 1998; Baines et al., 1999; Rohrbough and Broadie, 2002). Dorsal neuronal cell bodies are exposed using a large diameter (~20 µm) patch pipette containing 0.5-1% protease (type XIV (Sigma) in recording saline). The CNS sheath material is drawn by suction into the pipette tip for 1-2 minutes until the sheath ruptures (Featherstone et al. 1995). To confirm cellular identity during recordings, lucifer yellow (dipotassium salt, 1-2 mg/ml) can be added to the patch solution in order to visualize the position and morphology of the cell.
Standard whole-cell voltage- and current-clamp recordings can be made  (Rohrbough and Broadie, 2002; Featherstone et al., 2005). The external recording saline contains (in mM): 140 NaCl, 3 KCl, 2 CaCl_2_, 4 MgCl_2_, 5 HEPES, 10 sucrose, 2 NaOH (pH 7.2). The patch solution contains (in mM): 140 K-Acetate, 2 MgCl_2_, 0.1 CaCl2, 10 HEPES, 1.1 EGTA, 2 Na2-ATP, ~6 KOH (pH 7.2).  Voltage-clamp recordings are made at holding potentials of -60 mV at 18 °C.  Typical unadjusted resting potentials are in the range of -45 to –65 mV (-52.9 ± 7.5 mV, mean ± S.D., n = 78).
Available assays reveal voltage-gated ionic currents (Baines and Bate, 1998), repetitive action potentials, synaptically-mediated activity and agonist-gated responses (Baines et al., 2001). Synaptic input to motor neurons is recorded in the form of both fast AP-mediated fast excitatory synaptic currents (ESCs), and periodic, sustained (up to 1 sec duration) episodes of excitatory input occurring several times or more per minute (Baines et al., 1999; Baines et al., 2001).
Cell soma respond to iontophoretically applied acetylcholine (ACh) and the inhibitory transmitter GABA (Baines and Bate, 1998; Featherstone et al. 2005). ACh can be applied iontophoretically to the neuronal soma via a sharp microelectrode containing 100mM ACh in dH_2_0, at pH 4-5 to favor agonist iontophoresis by positive current.

### Representative Results

Ventral longitudinal muscle 6 is segments A2-A3 is the most commonly utilized target for recording (Broadie and Bate, 1993a-d; Harrison et al. 1994; Sweeney at al. 1995; Renden et al. 2001; Huang et al. 2006; Mohrmann et al. 2008). In the mature embryo and newly-hatched first instar larva (20-21 hrs AEL at 25°C = hatching), muscle 6 whole cell capacitance is typically 25-30 pF, and input resistances 20-40 MΩ, changing with developmental age. Maximal NMJ synaptic currents range from tens of picoamps (13-14 hrs AEL) to 1-2nA (20-21 hrs AEL), rapidly increasing as a function of age. Direct measure of glutamate-gated currents yield maximal responses of hundreds of picoamps (13-14 hrs AEL) to 1.5-4nA just prior to hatching. Series resistance errors (total current X series resistance) are usually <5 mV but, in mature, pre-hatching embryos, may be as high as 10+ mV and thus series resistance compensation is advised.  Embryonic muscles with an average diameter of 10-30 μm, and average length of 40-80 μm, appear to show nearly ideal space clamp parameters. Cell clamp time constants (R_series_ X C_cell_) average less than 0.25 msec.

In the mature embryo and newly-hatched larva, the dorsal motor neuron population has been subject to more limited recording. Neuronal soma recordings reveal whole cell capacitance in the range of 1.8-2.5 pF (2.0 pF average), series resistance of 40-60 MΩ and input resistance of ~5 GΩ (Baines and Bate, 1998; Featherstone et al 2005). These cells clearly grow in size and complexity during embryonic maturation, and this growth continues throughout larval development. In third instars, whole cell capacitance is 17-20 pF, series resistance is 25-37 MΩ and input resistance averages ~900 MΩ (Rohrbough and Broadie, 2002; Richard Baines, personal communication). Ionic conductances increase throughout development, but current density (pA/pF) remains remarkably constant. In mature embryos, synaptic currents average 75-100pA in amplitude, with both rapid ESC events and prolonged currents averaging ~500 msec (Baines and Bate, 1998; Featherstone et al 2005).  In embryos, these sustained events average 3-5 per min, increasing to ~20 per min in the first instar (Richard Baines, personal communication).  Note that the morphological complexity and membrane properties of *Drosophila* neurons makes it virtually impossible to properly voltage clamp much of the cell beyond the soma.  One should therefore interpret recordings with care.

## Discussion

Electrophysiological recording from *Drosophila* embryos requires manual manipulation and dissection. The health of the preparation, and consequent quality of the recordings, depends on one being able to quickly and neatly prepare the fragile embryonic tissues for recording, and then execute the experiment. Experimenters should be proficient in both embryonic dissections and patch clamp electrophysiology before trying to tackle both at once.

Recordings can be done in one of several variants of the “modified standard” or “haemolymph-like” (HL) salines. The primary differences between these two saline classes are 1) Na^+^ concentration (120-135 mM standard vs. 70 mM HL) and 2) Mg^2+^ concentration (4 mM standard vs. 20 mM HL). The major advantage of the high Na^+^ concentration in the standard solution is to facilitate action-potential propagation and, therefore, the maintenance of normal, patterned synaptic transmission. The HL salines suppress action-potential mediated synaptic transmission. The difference in Mg^2+^, which blocks voltage-gated calcium channels, alters the amplitude of synaptic transmission and shifts the Ca^2+^-dependency range upwards in the HL salines (Stewart et al. 1994). HL salines therefore show much lower synaptic transmission amplitudes at a given [Ca^2+^]. Careful control of osmolarity eliminates cellular vacuolation in both salines, and morphology appears equally well preserved in either saline. Since neither saline replicates actual haemolymph, the selection of a saline should reflect the needs of the question under study.

There are several important guidelines to maximize success of embryonic recordings. First, the ability to successfully form gigaohm seals on embryonic muscle decreases with developmental age. This is likely due primarily to development of the basement membrane surrounding the maturing muscles. To counteract this, longer collagenase treatment (1-2 min) is required. However, care must be taken because this lengthened enzymatic treatment will quickly compromise the muscle attachment to the body wall, and can easily lead to muscle detachment. One rule we have found effective is to limit the enzyme treatment to ~50% of the time that causes detectable muscle detachment. Second, it is crucial that the nerve has a tight fit in the suction electrode for adequate stimulation; too loose and the stimulation fails, too tight and it is not possible to stimulate an adequate section of the nerve. In our experience, this is definitely the biggest single limitation to successful NMJ recording. The appropriate diameter for the suction electrode must be empirically determined, but every effort should be made to preserve a good stimulation electrode, which can easily be used for an entire day of recording. Third, for successful neurotransmitter and drug applications to the NMJ, it is critical to become familiar with synapse placement. With experience, the stereotyped endings can easily be found with DIC optics. For the beginner, nerve terminals in the embryo can be revealed with membrane-permeant fluorescent mitochondrial dyes, such as Rhodamine 123 or 4-Di-2-Asp (Yoshikami and Okun, 1984; Broadie and Bate, 1993a). The dissected embryo is simply exposed to the dye (5mM, 5 mins) and excess dye removed with several washes of saline. The preparation is then viewed with an epi-fluorescence attachment and appropriate filters. Similar approaches can utilize transgenic GFP constructs marking the embryonic synapse.
